# Discrimination of semantically similar verbal memory traces is affected in healthy aging

**DOI:** 10.1038/s41598-024-68380-0

**Published:** 2024-08-02

**Authors:** Alex Ilyés, Borbála Paulik, Attila Keresztes

**Affiliations:** 1 Doctoral School of Psychology, ELTE Eötvös Loránd University, Budapest, Hungary; 2 Brain Imaging Centre, HUN-REN Research Centre for Natural Sciences, Budapest, Hungary; 3 Institute of Psychology, ELTE Eötvös Loránd University, Budapest, Hungary

**Keywords:** Mnemonic discrimination, Aging, Semantic similarity, Pattern separation, word2vec, Human behaviour, Ageing, Learning and memory

## Abstract

Mnemonic discrimination of highly similar memory traces is affected in healthy aging via changes in hippocampal pattern separation—i.e., the ability of the hippocampus to orthogonalize highly similar neural inputs. The decline of this process leads to a loss of episodic specificity. Because previous studies have almost exclusively tested mnemonic discrimination of visuospatial stimuli (e.g., objects or scenes), less is known about age-related effects on the episodic specificity of semantically similar traces. To address this gap, we designed a task to assess mnemonic discrimination of verbal stimuli as a function of semantic similarity based on word embeddings. Forty young (M_age_ = 21.7 years) and 40 old adults (M_age_ = 69.8 years) first incidentally encoded adjective-noun phrases, then performed a surprise recognition test involving exactly repeated and highly similar lure phrases. We found that increasing semantic similarity negatively affected mnemonic discrimination in both age groups, and that compared to young adults, older adults showed worse discrimination at medium levels of semantic similarity. These results indicate that episodic specificity of semantically similar memory traces is affected in aging via less efficient mnemonic operations and strengthen the notion that mnemonic discrimination is a modality-independent process supporting memory specificity across representational domains.

## Introduction

Human memory is continuously changing across the lifespan with various memory models^1^ positing specific trajectories for different memory systems. For instance, episodic memory is prone to a steeper age-related decline compared to semantic memory^1–4^. However, comparisons of episodic and semantic memory decline are confounded by the fact that studies of episodic memory predominantly assess cognitive processes (e.g., recalling information), whereas studies of semantic memory predominantly assess quality of representations (e.g., vocabulary size)^5^.

A way to address this confound is to focus on a single cognitive process while manipulating representational demand, assessing the sensitivity of the process to various representational modalities^6^. One key cognitive process impacted by aging is mnemonic discrimination^7–9^, i.e., the ability to maintain highly specific episodic memory traces. Age-related decrease in mnemonic discrimination potentially underlies decreased specificity of memories in old age^10–12^. We selected this process because it is often considered the behavioral correlate of a single neural operation (i.e., hippocampal pattern separation)^13,14^, yet it is unclear whether it can be generalized to other representational modalities because it has been almost exclusively tested via visuo-perceptual representations. Specifically, it is unclear whether age affects discrimination of semantically similar memory traces.

A widely-used tool to assess mnemonic discrimination is the Mnemonic Similarity Task (MST)^15,16^. In a typical test, participants encode pictures of objects or scenes and later, their recognition performance is assessed for pictures that are identical or highly similar to the encoded ones. One recent study^17^ using an MST-like design, modeled both visual and semantic similarity of pictures representing objects, and probed recognition performance of both young and old adults. They found that older adults compared to younger adults relied on semantic instead of perceptual information to correctly identify similar lures. Even though this result suggests a spared usage of semantic information for episodic specificity in aging, it is hard to reconcile with findings suggesting that a stronger representation of semantic ‘gist’ in old adults often impact memory for specific details too^18,19^. This apparent tension might arise from a cross-modal modeling of semantic similarity of visuo-perceptual stimuli.

To our knowledge the only investigation of mnemonic discrimination in purely the conceptual domain in aging^20^ assessed mnemonic discrimination of words that were similar to each other either conceptually (such as ‘baby’ and ‘rattle’) or phonologically (e.g., ‘curtain’ and ‘certain’). The results suggested an age-related deficit in perceptual but not conceptual mnemonic discrimination. Importantly, this study had two features that prevented a direct comparison across perceptual and conceptual mnemonic discrimination. First, it did not parametrically manipulate conceptual similarity, and second, conceptually similar words often violated category boundaries, introducing different task demand for conceptual versus perceptual discrimination.

For our study, we designed a recognition task that addressed the above challenges and allowed us to manipulate conceptual similarity akin to manipulations of perceptual similarity in extant studies of mnemonic discrimination. Instead of single words, here we used adjective-noun phrases as a to be encoded target stimuli (e.g., ‘wired microphone’) and manipulated only the adjective when creating conceptually similar lures (e.g., ‘desktop microphone’). This manipulation is similar to the original MST^16^, where participants see objects (e.g., a red apple) as targets, and the same object with different perceptual features as lures (e.g., reddish green apple). The shared noun across semantically similar phrases also ensured that targets and lures were always from the same conceptual category. Importantly, we used a context-sensitive word2vec word-embedding^21^ to manipulate conceptual similarity between adjective-noun phrases^22,23^ in an incidental recognition task similar to the MST. This design allowed us to assess the cognitive process of mnemonic discrimination as a function of conceptual similarity without confounds arising from visuo-perceptual similarities. Using this design, we aimed to reveal age-related differences in processing otherwise intact semantic representations.

## Methods

### Transparency and openness

Sample size, study design, hypotheses and analysis plans were preregistered on Open Science Framework (OSF)^24^, and link to access is provided in the Data availability statement. We report how we determined our sample size, any data exclusions, all manipulations, and all measures in this study.

All experimental procedures and materials were reviewed by the Research Ethics Committee at the Faculty of Education and Psychology at Eötvös Loránd University (permission number: 2022-02), and all participants gave informed written consent. All research was conducted in conformity with the Declaration of Helsinki.

All materials, all de-identified data on which the study conclusions are based, and all analytic code needed to reproduce analyses are available online, and link to the access is provided in the Data availability statement.

All analysis were conducted in R programming language (version 4.2.1)^25^ with the use of the ‘tidyverse’ package (version 1.3.2)^26^ for data cleaning, the ‘ggplot’ (version 3.4.0)^27^, the ‘sjPlot’ (version 2.8.12)^28^, and the ‘smplot2’ (version 1.0)^29^ packages for creating figures, the ‘psycho’ package (version 0.6.1)^30^ for calculating d’ scores, and the ‘lme4’ package (version 1.1.30)^31^ for mixed effects modeling.

### Participants

Between April and September 2022, forty young (*M*_*age*_ = 21.7 years*, SD*_*age*_ = 2.03, 30 females, all Caucasian) and forty older adults (*M*_*age*_ = 69.8 years*, SD*_*age*_ = 3.98, 32 females, all Caucasian) took part in the experiment in exchange for course credits (young adults) and vouchers (older adults) at our lab (Research Centre for Natural Sciences, Budapest, Hungary) in an urban area. Sample size was determined based on a power analysis conducted in G-Power 3^32^ for a Wilcoxon-Mann–Whitney test comparing the d’ value of two age groups (old [n = 18] and young [n = 21] adults) on a mnemonic discrimination test with verbal stimuli, yielding an effect size (Cohen’s *d)* of 0.85 and a* p* of 0.027^20^. We note that Ly and colleagues found a significant effect of phonetic—and not conceptual—similarity on mnemonic discrimination and they only tested age effects, however for lack of a better estimate, this was the effect we based our power calculations on. The power calculation yielded a necessary sample size of 39 for both age groups, but we decided to include 40 participants to have a multiple of four participants to ensure the same number of individual responses across all four stimulus conditions for the stimulus-based fixed effects in our multiple regression model (see ‘Randomization’ section below).

Young adults were recruited through a course at Eötvös Loránd University, Budapest, Hungary, whereas older adults were recruited via social media and databases of the Research Centre of Natural Sciences, Budapest, Hungary. Participants did not have a medical history of neurological, psychological, or cardiovascular diagnosis, had normal or corrected-to-normal vision, and had at least a high-school degree. Older adults were additionally screened by the Montreal Cognitive Assessment (MoCA)^33,34^ with a 23-point cut-off to ensure normal cognitive status^35^. We recruited two additional participants after excluding two participants based on MoCA scores. In the final sample, all 40 older adults scored above 23 points on the MoCA test (*M* = 25.95*, SD* = 2.01).

### Materials

#### Main task (Semantic mnemonic similarity task)

**Figure 1:**
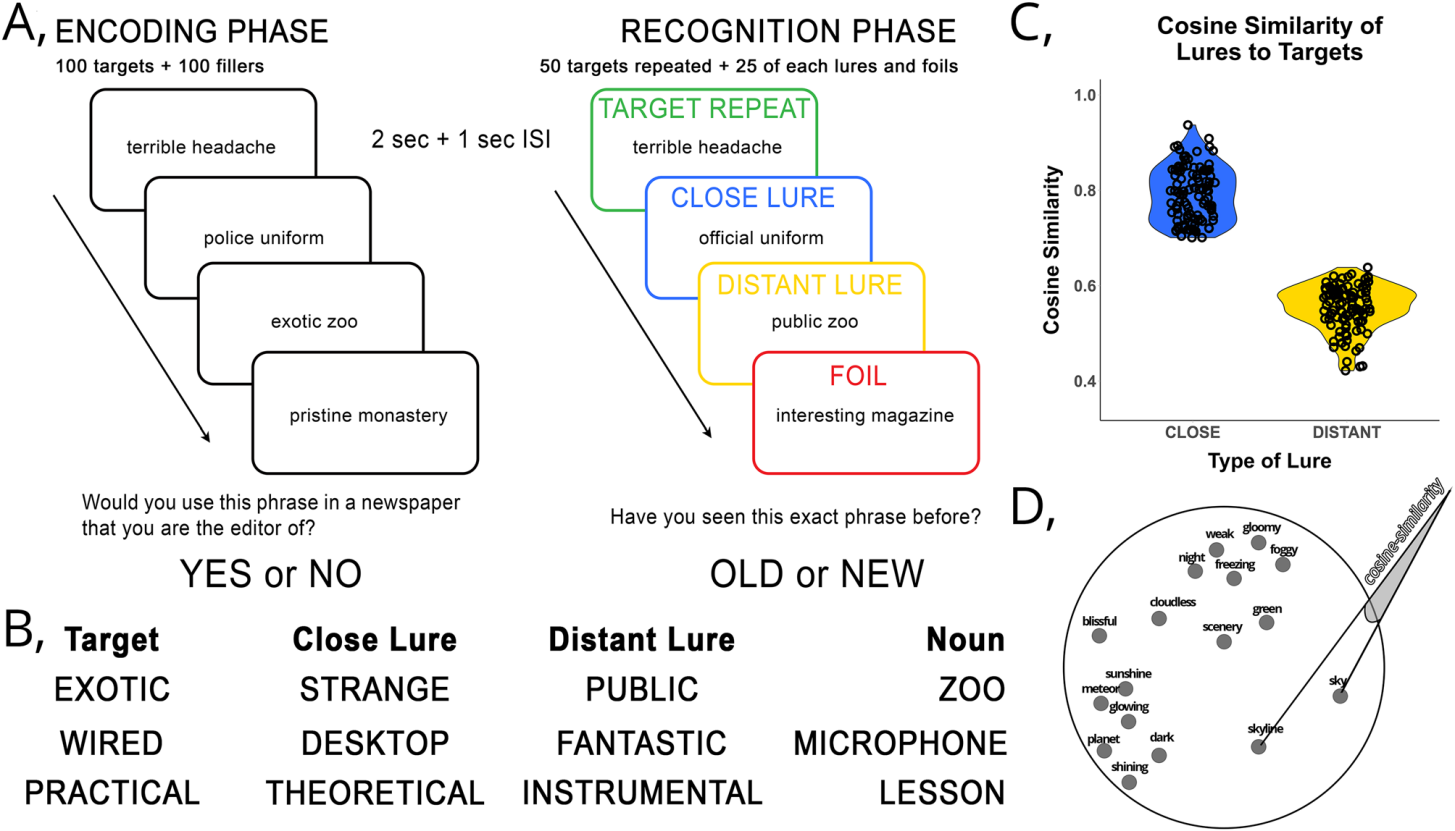
Main methodological components of our main task, the sMST. (**A**) The design of the sMST. Participants saw 200 adjective-noun phrases in an incidental encoding phase, and they had to decide whether they would allow such phrases in a magazine they are the editor of (‘yes’ vs ‘no’). After a 15-min break with an intermittent 1- and 2-back task, participants saw 125 phrases (50 exact repeats, 50 lures and 25 foils). They had to decide whether they had seen the exact phrase before (‘old’ vs ‘new’). (**B**) Example phrases from the sMST stimuli. We made 300 phrases with 100 nouns that were shared between target, close lure, and distant lure conditions. Target adjective was the baseline, close lures were close in the semantic space, while distant lures were distant. (**C**) Cosine similarity was calculated from the Hungarian word2vec^37^ based on adding up the vector for the noun and the adjective. Each close lure (blue) had a higher cosine similarity rating than 0.7 with its target pair, and each distant lure (yellow) had a lower cosine similarity rating than 0.65 with its target pair. (**D**) Two-dimensional visual representation of the semantic neighborhood of ‘sky’ in word2vec. Notice how similar themed words are clustered in the same segment.

We developed this task following the design of the incidental version of the MST^36^. The semantic Mnemonic Similarity Task (sMST; Fig. 1A) uses a verbal stimulus set of phrases to test recognition memory and mnemonic discrimination of targets and semantically similar lures. In contrast to earlier work^20^ using single nouns as verbal stimuli in a similar design, we decided to use adjective-noun phrases and to manipulate the similarity by highlighting different features of the same concept. For instance, in the sMST ‘exotic zoo’ is a target and ‘strange zoo’ is a semantically similar lure, both sharing the same concept, and different only in a semantic detail. This is analogous to the commonly used perceptual version of the MST, where targets and similar lures are pictures of the same object concept with different perceptual features (e.g., a glass full of water, and a similar glass half empty). The use of noun-adjective phrases in this way also ensured that we did not violate category boundaries as in Ly et al.^20^ where a concept from a category (e.g., ‘rattle’ being an object) could be a conceptual lure for a concept from an entirely different category (e.g., ‘baby’ being a living entity), which may have dampened any existing mnemonic similarity effects.

##### Stimuli

Stimuli consisted of 100 target phrases (e.g., ‘exotic zoo’), 100 closely related semantic lures (e.g., ‘strange zoo’; close lures), 100 distantly related semantic lures (e.g., ‘public zoo’; distant lures, Fig. 1B), and 125 novel foils (e.g., ‘relevant advertisement’), generated from 225 nouns and 425 adjectives (100 nouns paired with three different adjectives, and 125 nouns paired with one adjective each).

To select the words and to measure semantic similarity parametrically, we used a Hungarian word2vec^21^ word-embedding^37^ created based on the ‘press’ subcorpus of the Hungarian National Corpus (HNC)^38^. The corpus consists of collected transcribed, online, and printed press texts with approximately 364.8 million words from 2004 until 2012. Algorithms predicting either the context of given words or words of a given context were used to derive a 200-unit-long numerical vector for each word by another group^37^. These numeric vectors offer a way to mathematically compare how similar two words are, based on the similarity of their context^39^. The most utilized metric for measuring similarity in a word2vec word-embedding is cosine similarity $\left( {\frac{A \cdot B}{{\left\| A \right\|\left\| B \right\|}}} \right)$ where A and B are word vectors, and their dot product is divided by the product of their L2-norms, illustrated on Fig. 1D), which compares the angle between two vectors, giving a robust estimate of similarity in large dimensionality. According to the authors^21^ vectors in this space encode multiple linguistic patterns and regularities, thus their similarities do not simply reflect synonymous meaning, but other syntactic and pragmatic factors too, such that the model is also able to reliably predict analogies (e.g., subtracting the vector of ‘man’ from the vector of ‘king’ and adding the vector of ‘woman’ results in a vector most similar to the vector of ‘queen’). Since, we are comparing combined meanings of adjective-noun phrases, we summed the adjective and noun vectors to create a compound vector and calculated the cosine similarity between each compound vector. This method is sensitive to aggregated meaning representation and is not statistically worse than more sophisticated concept vector aggregations^40^.

To ensure that the stimuli set was as homogeneous and meaningful as possible, we adhered to the following procedure: We first filtered the complete subcorpus for words with only one meaning that can function as only one part of speech (either adjective or noun). We then restricted our selection to predefined ranges of grammatical variables known to bias memory decisions^41^. We filtered based on frequency (35–90 percentiles) from the HNC^38^, character count (7**–**13 characters), syllable count (2**–**6 syllables, Supplementary Fig. 3) and phonological neighborhood (ensuring that no word had more than 5 phonological neighbors, i.e., words that share all but one character). This resulted in 1164 adjectives and 1515 nouns. After initial filtering, two experimenters (A.I. and B.P.) selected 225 nouns they subjectively rated as the most concrete and ordinary. Experimenters reviewed, discussed, and made joint decisions on inclusion of nouns they disagreed on the inclusion of. Sensitive (e.g., ‘fascism’) jargon (e.g., ‘misappropriation’), and too unordinary (e.g., ‘intensity’) words were the most often excluded.

In a second step, each noun was combined with each of the 1164 adjectives and the 10 most meaningful phrases were selected for each noun (e.g., 'exotic zoo' is meaningful in contrast to 'humorous zoo', which is not). This was a semi-automated process, as we calculated the pairwise cosine similarity of each adjective with each noun, sorted them in a descending order, and selected the 10 most meaningful from the top of the list based on subjective decision. Because cosine similarity is sensitive to semantic and contextual similarity, this appeared as a good proxy for adjective-noun fit. Then, each of the 10 phrases for a single noun were compared to all the other adjective and noun combinations (1163 remaining adjectives), and cosine similarity was calculated between all of them. Based on a matrix of cosine similarities for each word, the two experimenters selected one target phrase from the 10 most meaningful phrases, then selected one lure phrase that was semantically close (had a cosine similarity of 0.7 or above) and one lure phrase that was semantically distant (had a cosine similarity between 0.4 and 0.7, Fig. 1C). Because cosine similarity is agnostic to the pragmatic fit between words, when their joint compounds were constructed some phrases were deemed nonsense for human readers (for instance, both words in the phrase ‘musical guitar’ share the same semantic context, but the phrase would never be used in human speech because “musical” is redundant in it). In some cases, when an already selected adjective seemed to be a better fit for a new noun, the two experimenters needed to reach an agreement whether to change a triplet that was already selected, to be able to use the adjective in another triplet. For example, ‘humorous professor’ had been already selected as a distant lure for ‘economist professor’ when upon searching for a close lure for ‘comical play’, the adjective ‘humorous’ was deemed to be a better close lure paired with ‘play’, than a distant lure paired with ‘professor’.

After creating 100 target—close lure—distant lure triplets, the experimenters used the remaining nouns and adjectives to create 125 foils, keeping in mind that adjectives semantically close to target adjectives should not be used (above 0.7 cosine similarity).

Finally, in an independent sample 30 young (*M*_*age*_ = 22.56*, SD* = 6.37, females = 24) and 34 older adults (*M*_*age*_ = 71.18*, SD* = 5.1*,* females = 18), all Caucasian) , we assessed each of the 425 phrases on psycholinguistic measures potentially affecting word recognition memory: concreteness^42^, arousal^43^ and meaningfulness^44^, using a 7-point Likert scale (e.g., ‘How concrete is this phrase?; 1—very abstract, 7—very concrete’) For each construct we measured, we gave an example phrase to participants (e.g. ‘”delicious bread” is a highly concrete phrase, because you can easily experience the object it denotes, with your senses, while “ambiguous morality” is highly abstract, because you cannot experience it with your senses’) as practice. For each questionnaire, we randomly split the 125 foils into three bins of 41, 42, 42 bins and assigned each bin to either the 100 targets, 100 close lures or 100 distant lures to ensure that no noun is repeated in a single questionnaire. Participants then rated each phrase in the questionnaire on all three constructs. This resulted in 10 data points on each scale for all phrases in both age groups (Supplementary Fig. 3). We conducted two-way ANOVAs for each covariate measure with age group and condition as independent variables. Results are summarized in Supplementary Table 2.

##### Design and procedure

We used a mixed design with age group (young adults, old adults) as a between-subject variable, and stimulus condition (target, close lure, distant lure, foil) as a within-subject variable.

In an incidental encoding phase, participants viewed 200 adjective-noun phrases. Phrases were either targets (100) which were manipulated or repeated in a later recognition phase; or fillers (100) which did not appear later at recognition. Participants were instructed to pretend that they were the editors of a newspaper, and for each phrase they needed to decide whether they would allow them to go in print. This cover story was implemented to focus participants’ attention to the meaning, and to facilitate processing of conceptual information and similarity. In each trial, a fixation cross appeared in the middle of the screen for one second followed by a phrase shown on the middle of the screen for two seconds with available response options (via a button box) of ‘blue—yes, I would use this phrase’ written in the bottom left and ‘red—no, I would not use this phrase ‘ written in the bottom right corner.

A recognition phase followed after a five-minute break and a 10-min distractor n-back task (see below). Here, participants viewed 125 adjective-noun phrases. Phrases were either exactly repeated targets (same noun, same adjective; n = 50), novel foils (novel noun, novel adjective; n = 25) or lures (same noun, novel adjective, n = 50). Lures were either ‘close lures’ (n = 25) or ‘distant lures’ (n = 25) as a function of their semantic distance from their target (see ‘Stimuli’). Participants were instructed to decide whether they had seen the exact same phrase in the encoding phase. In each trial, a fixation cross appeared in the middle of the screen for one second followed by a phrase shown in the middle of the screen for two seconds with the response options ‘blue—I have seen this’ written in the bottom left and ‘red—I have not seen this’ written in the bottom right corners. Next, a one-second inter-stimulus-interval (ISI) followed, during which late responses were registered. Following each recognition decision, participants were asked to indicate how confident they were in their decision. They saw a screen with only three response options displayed (‘blue—guessing’, ‘yellow—unsure’, and ‘red—sure’ on the bottom left, middle, and right side of the screen, respectively) for 15 s or until they responded, and were instructed to press the button corresponding to their confidence judgment.

Participants practiced six trials of both the encoding phase and the recognition phase.

##### Randomization

Participants were pseudorandomly assigned unique stimulus lists to collect the same number of responses for all phrases of each stimulus condition in the total sample. This allowed us to define random-effects for items in a multivariate Generalized Linear Mixed-Effects Model. Having unique randomized lists for all participants also allowed for controlling for potential list order effects. Adjective-noun phrases in a stimulus list were pseudorandomized with the constraint that a maximum of three consecutive trials could be drawn from identical stimulus conditions.

During randomization, we made sure to rotate item lists across participants such that each stimulus was presented equal number of times in its own condition. As a result, 40 participants provided 10 data points for each close and distant lure, 20 data points for each target, and 8 data points for each foil. We provide further information on our randomization process in the Supplementary Material (section Randomization).

##### N-back

An n-back task with two blocks (a 1-back followed by a 2-back) of 25 trials served as a distractor between encoding and recognition in the sMST. In each trial, participants viewed a letter sampled from a pool of 10 letters (D, F, H, L, P, Q, Y, Z, S, J) in the middle of the screen for three seconds (ISI = 1 s) or until response. Ten trials per block were targets, i.e., letters appearing in trials prior. Participants were instructed to press a designated button on a button box when they detected a target.

#### Covariate assessments

Participants completed a batch of covariate tasks and questionnaires to allow us to control for confound variables known to affect memory in healthy aging, including cognitive status^45^, non-word repetition^46^, depressive symptoms^47–49^, lifestyle^50,51^, processing speed^52,53^ and vocabulary^54^.

To assess phonological awareness and verbal working memory we administered a non-word repetition task^55^, Hungarian adaptation:^56^. Depressive symptoms were assessed by the Beck Depression Inventory^57^, Hungarian adaptation:^58^. To collect data about the health and lifestyle of participants, they filled out an in-house questionnaire (see Supplementary Table 1) assessing variables frequently related to age-related memory decline^59–66^. We also administered the original version of the Mnemonic Similarity Task^15,36^, and we used two subtests from the Hungarian Wechsler Adult Intelligence Scale (WAIS-IV)^67^, to assess processing speed (Digit-Symbol Substitution Test) and crystallized knowledge (Vocabulary Test). For further details see the Supplementary Material.

### Overall procedure

Participants completed all tasks in a closed, windowless lab with two tables—one where the paper and pencil tests were administered, and one with a desktop computer set up for the experimental tasks. Responses for all computer-based tasks were recorded on a CEDRUS KB-740 button box with seven buttons in a horizontal line, except for the Mnemonic Similarity Task, for which participants used a QWERTY keyboard. The button box had three colored buttons which were used during the experiments: the second from the left (blue), the middle button (yellow), and the second from the right (red). The rest of the buttons were left as plain white and were not used or registered during any tasks.

Upon arriving at the lab, participants signed the informed consent and completed a checklist for inclusion criteria. Then, elderly participants completed the MoCA. All other tests were then administered for both age groups in the same order: First, participants completed the sMST, followed by a 10–15 min break. Then, they completed all covariate assessments in the order listed in the ‘Covariate assessments’ section. Short breaks were introduced between tasks based on participants’ demands.

Participants received remuneration (5000 HUF ~ 13 EUR) after completing all tasks.

### Statistical analyses

To assess mnemonic discrimination in the sMST, we calculated a d’ value that expresses the ability to discriminate between two stimulus conditions. This metric used in MST tasks with two response options ('old' vs 'new') is similar to the Lure Discrimination Index (LDI) calculated in MST tasks with three response options ('old', 'similar', 'new'; see: Stark, 2019). D’ for targets vs. lures (lure discriminability) was calculated as the z-transformed proportion of hits (‘old’ responses to targets) minus the z-transformed proportion of false alarms to lures (‘old’ responses to lures), i.e., z(p(‘old’|target))—z(p(‘old’|lure)). D’ for targets vs. foils—foil discriminability, which is similar to a traditional recognition score –is also calculated as the z-transformed proportion of hits (‘old’ responses to targets) minus the z-transformed proportion of false alarms to foils (‘old’ responses to foils), i.e., z(p(‘old’|target))—z(p(‘old’|foil)).

To compare the performance of older vs. younger adults in discriminating lures and foils from targets, we ran a two-way mixed-effects ANOVA on the d’ scores with a between-subject factor of age group (old adult vs. young adult) and a within-subject factor of stimulus condition (target vs. close lure, target vs. distant lure, target vs. foil). To assess age-differences in mnemonic discrimination while also controlling for item- and participant-level covariates, we modeled the response for recognition phrases (‘old’ vs. ‘new’) using Generalized Linear Mixed-Effects Modelling (GLMEM) based on fixed- (factor variables) and random-effect terms (ordinal and scale variables). Fixed effects of stimulus condition (target, close lure, distant lure, foil) and age group (old adults, young adults) were used with random effects specified for trial order, stimulus (item number) and participant (participant ID). Item-level covariates (frequency, syllables) were entered in interaction with the fixed effect term for stimulus condition. Participant-level covariates (non-word span, raw number of non-words repeated, LDI, Beck score, health and lifestyle variables, vocabulary score, processing speed, MoCA score) were entered in interaction with the fixed effect term for participant. Finally, psycholinguistic item-level covariates (concreteness, arousal, and meaningfulness) assessed by independent samples from both age groups were entered in a three-way interaction with stimulus condition and age group. Care was taken not to violate multicollinearity by checking the variance inflation factor (VIF) in our final model. If two or more variables were collinear, then we omitted one of the correlating covariates from our final model. We report *estimates* of the GLMEM that index the effects of contrasts on the likelihood of responding ‘old’ with an intercept of foil and young adults, such that an estimate > 1 signifies a positive change in likelihood, while and estimate < 1 signifies a negative change. We further elaborate on findings when necessary.

To assess the effect of semantic similarity on a continuous scale, we additionally defined a GLMEM where—instead of the categorical binning of lures—we modeled the response (‘old’ vs. ‘new’) with a fixed effect of semantic similarity as a continuous predictor (i.e., cosine similarity) and another fixed effect of age group (old adults, young adults).

On top of our preregistered analysis, to explore the relationship between mnemonic discrimination of concepts and that of pictures of everyday objects, we also assessed the association between mnemonic discrimination performance on the sMST and the MST.

## Results

### Mnemonic discrimination varied as a function of semantic similarity and age

**Figure 2:**
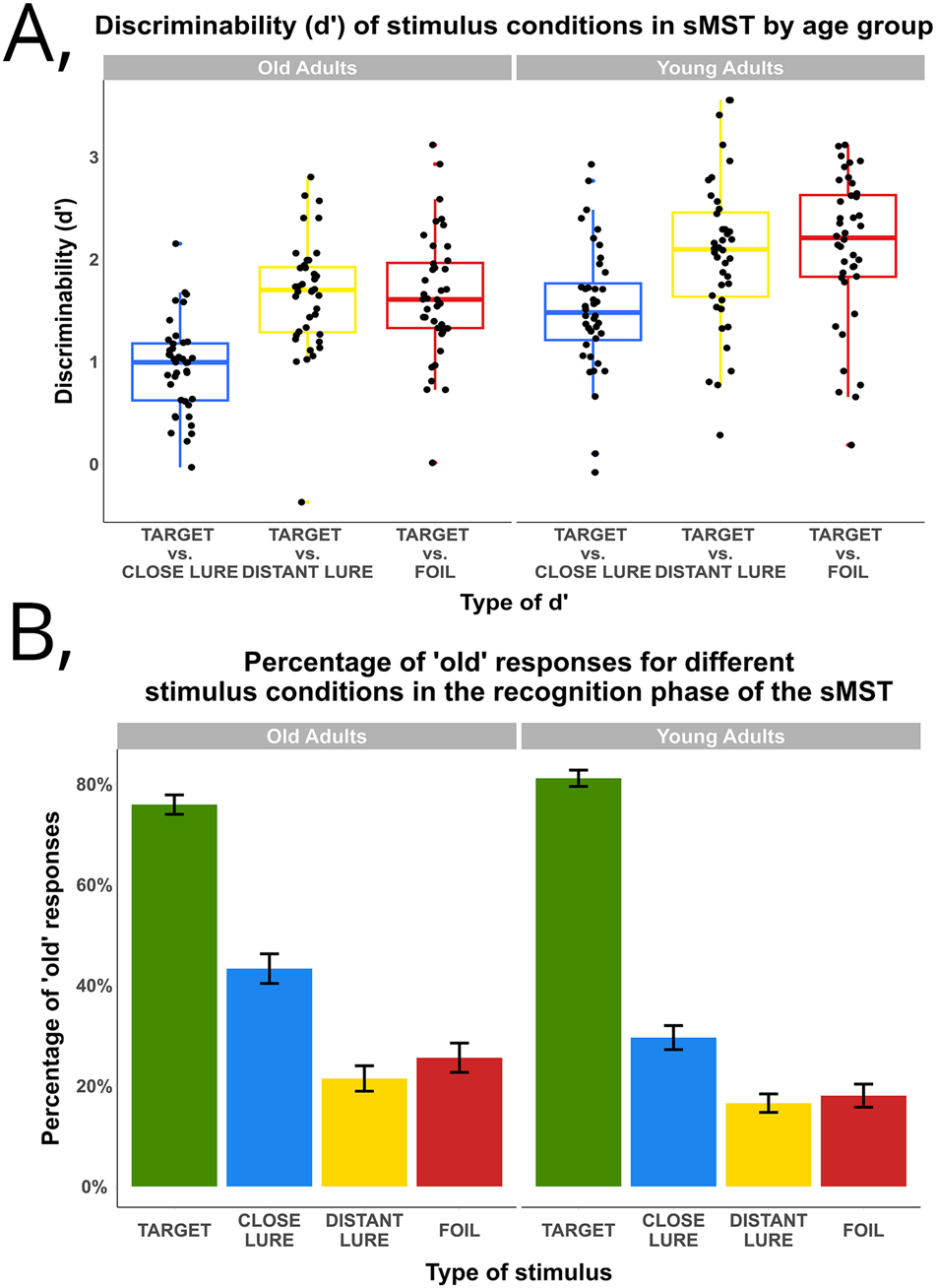
Main results of the sMST. (**A**) D’ scores in the sMST, derived from the z-transformed proportion of hits and false alarms on different contrasts of conditions. D’ scores show the discriminability of the two stimulus types in the respective contrast. The x-axis denotes the contrasts, while the y-axis shows the d’ score itself, faceted by the age groups. (**B**) Percentage of ‘old’ responses in the recognition phase of the sMST. ‘Old’ response is correct in case of targets, and in correct in case of any lures and foils. The x-axis shows the four stimulus types in the recognition phase of the task, while the y-axis shows the percentage of ‘old’ responses, faceted by age groups. Error bars represent the standard error.

For comparibility to^20^, we ran a two-way mixed analyses of variance (ANOVA) on d’ scores with stimulus condition as a within subject variable (target vs. foil, target vs. close lure, target vs. distant lure) and age group (old adults vs. young adults) (Fig. 2A).

Normality tests of d’ distributions using a Bonferroni-corrected Shapiro test (devised to test normality of multiple measures within the same sample), showed significant deviations from the normal distribution for distant lure vs. foil in older adults (*statistic* = 0.93*, p* = 0.01) and target vs. foil young adults (*statistic* = 0.93*, p* = 0.02) d’ scores. However, there were no extreme outliers above 3 SD, and removing two participants to ensure normality, more violations of normality were introduced in other measures. Thus, based on further inspection of Q-Q plots for each measure, we decided to leave the sample intact, because ANOVA is robust to minor deviations from normal distribution in measures of factors^68^. Levene’s test revealed no violation of homogeneity of variances in any conditions (all *p*s > 0.1).

In line with^20^ this ANOVA yielded a significant main effect of stimulus condition (*F (2, 234)* = 27.43*, p* < 0.001*, η2* = 0.17), confirming the presence of a semantic similarity effect on mnemonic discrimination performance in both age groups. However, contrary to^20^, we also found an effect of age group (*F (1, 234)* = 35.12*, p* < 0.001*, η2* = 0.11) on semantic similarity based mnemonic discrimination performance. Finally, we found no stimulus condition X age group interaction (*F (2, 234)* = 0.37*, p* = 0.69*, η2* = 0.002).

One limitation of using d’ in an ANOVA as a proxy for mnemonic discrimination is that we introduce a distortion by only looking at pairs of stimulus conditions and not the stimulus conditions themselves. Importantly, this analysis also precluded testing the effect of semantic similarity on mnemonic discrimination on a continuous scale, because for each participant d’ was calculated for a pair of stimulus conditions aggregated across phrases. These limitations had motivated us to conduct a generalized linear mixed modeling (GLMEM) analysis introduced next.

### Age interacted with discrimination between targets and foils

We used GLMEM to predict the type of response (‘old’ vs. ‘new’) for each stimulus condition (target, close lure, distant lure, foil) and age group (young adults, old adults, Fig. 2B), while controlling for participant-level depression score, vocabulary score, processing speed, non-word repetition, mnemonic discrimination of pictures, sleep score, exercise score, Supplementary Fig. 4), and item-level (concreteness by old adults, concreteness by young adults, arousal by old adults, arousal by young adults, noun frequency) covariates, and random effects of participant, trial order and item. We used ‘probit’ regression from the lme4 package^69^ following previous work^70^ where authors modeled the response differences for lures, targets and foils in the traditional MST task with different perceptual and conceptual control variables as predictors.

We first used Mahalanobis distance to establish the covariate structure of all the candidate data points for modeling fixed effects and interactions. We used χ2 *(2)* = 0.95 as a cut-off value for data points that were significant outliers. Based on this, we excluded four participants, two from each age group, and ran GLMEM on a sample of 38 young (*M*_*age*_ = 70*, SD* = 4.01) and 38 old adults (*M*_*age*_ = 21.6*, SD* = 2.03). Residual plots indicated that caffeine consumption, adjective frequency, phonological neighborhood, and meaningfulness by both age groups markedly deviated from the normal distribution. We thus decided to omit these variables from further analysis.

**Figure 3:**
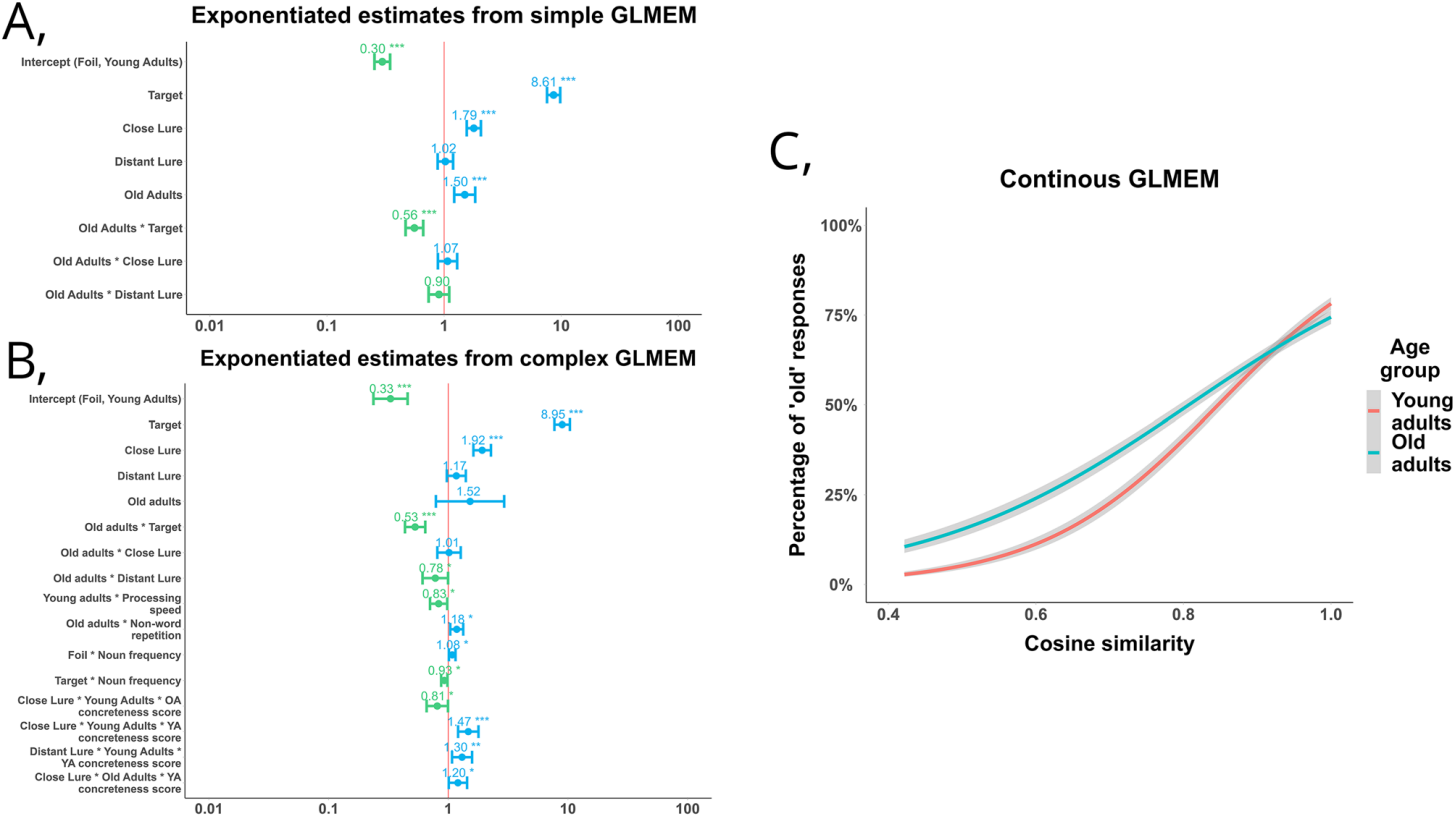
GLMEM results. (**A**) Exponentiated estimates, standard errors, and p-value of the fixed effects in the simple GLMEM predicting ‘old’ responses at recognition, based on the fixed and interaction terms of age group (young adults, old adults) and condition at recognition (targets, close lures, distant lures, foils). The intercept is foils and young adults. Estimates lower than 1 correspond to a negative effect, while estimates above 1 correspond to a positive effect on the probability of ‘old’ responses. (**B**) Exponentiated estimates, standard errors, and p-value of the fixed effects in the complex GLMEM predicting ‘old’ responses at recognition, based on the fixed and interaction terms of age group (young adults, old adults) and condition at recognition (targets, close lures, distant lures, foils), plus the interaction of all he covariates with the respective fixed effect (participant-level covariates with age group, item-level covariates with condition). The intercept is foil and young adults. Only significant estimates of covariate interactions are shown. Estimates lower than 1 correspond to a negative effect, while estimates above 1 correspond to a positive effect on the probability of ‘old’ responses. (**C**) Percentage of ‘old’ responses for targets, close lures, and distant lures as a function of cosine similarity measured with the word2vec model with red line showing young adults and green line showing old adults. Young adults compared to old adults show a steeper increase in false alarm rate with increasing similarity.

First, we defined a simple model, with only the two predictors as fixed effects and in interaction: age group and stimulus condition predicting old vs. new responses at recognition (Fig. 3A). We defined baseline levels as (a) foil for stimulus condition to see whether lures were identified as ‘old’ more likely than foils, and (b) young adults for age groups. The model had a good fit (*AIC* = *9048.8*, *conditional R*^*2*^ = 0.47*)*. Main effects were significant for close lure (*estimate* = 0.56*, SE* = 0.04*, p* < *0.0*01*, CI* = 0.49–0.65), and target (*estimate* = 8.61*, SE* = 0.57*, p* < *0.0*01*, CI* = *7.56*–*9.8*), but not for distant lure (*estimate* = 1.02*, SE* = 0.08*, p* = 0.75*, CI* = 0.88–1.19), indicating that all participants were more likely to incorrectly recognize close lures, but not distant lures compared to foils, and that participants were more likely to respond ‘old’ to targets than to lures replicating our ANOVA findings. The main effect of age was also significant (*estimate* = 1.5, *SE* = *0.1*6, *p* < 0.001*, CI* = 1.22–1.84) mirroring the fact that older adults overall performed worse than young adults. Finally, this GLMEM revealed a significant target x old adults interaction (*estimate* = 0.56*, SE* = 0.05, *p* < 0.001*, CI* = 0.47–0.66) indicating that older adults were less likely to correctly respond ‘old’ to targets compared to foils than young adults.

### Older adults were less likely to false alarm for distant lures compared to foils

Second, we defined a complex model, with stimulus condition and age group in interaction and covariates as interaction terms with their respective fixed effects (participant-level covariates with age group, noun frequency with stimulus condition, and psycholinguistic item-level covariates in a three-way interaction with both stimulus condition and age group, Fig. 3/B). This model had a better fit (*AIC* = 9026.7*, conditional R*^*2*^ = 0.48*)*, than the simple model (χ2 *(50)* = 144.06, *p* < 0.001). Stimulus condition main effects were significant for close lure (*estimate* = 1.92*, SE* = 0.17*, p* < 0.001, *CI* = 1.62–2.28), and target *(estimate* = 8.95*, SE* = 0.68, *p* < *0.0*01*, CI* = 7.72–10.4) but not for distant lures (*estimate* = 1.17, *SE* = 0.11*, p* = 0.09*, CI* = 0.97–1.4), indicating that compared to foils, close—but not distant—lures were likely to be incorrectly identified as ‘old’. The age effect was not significant (*estimate* = 1.52*, SE* = 0.51*, p* = 0.21, *CI* = 0.6–3.46) indicating that age-related differences in mnemonic discrimination were at least in part accounted for by age-difference in covariates. However, this model revealed a significant distant lure x old adults interaction (*estimate* = 0.78*, SE* = 0.1*, p* = 0.045, *CI* = 0.61–1.0) indicating that in contrast to young adults (and our expectations), older adults were more likely to incorrectly respond ‘old’ to foils than to distant lures, even though distant lures feature repeated concepts.

A look at interactions of participant-level covariates revealed a significant young adults x processing speed (*estimate* = 0.83*, SE* = 0.07*, p* < 0.01*, CI* = 0.7–0.99) interaction indicating that young adults who have faster processing speed, responded ‘old’ incorrectly less often for foils in the sMST. Additionally, we found an old adults x non-word repetition interaction (*estimate* = 1.18, *SE* = 0.07*, p* = 0.01*, CI* = 1.04–1.33) surprisingly showing that old adults who were able to repeat longer non-words responded ‘old’ incorrectly more often to novel foils in the sMST. Crucially, vocabulary as measured by the WAIS subtest did not have a significant effect in the model whatsoever (Vocabulary x old adults: *estimate* = *0.96, SE* = *0.57, p* = *0.06, CI* = *0.85*–*1.1*; Vocabulary x young adults: *estimate* = *0.91, SE* = *0.06, p* = *0.16, CI* = *0.8*–*1.04*). Effects of item-level covariates are detailed in the Supplementary Material (Supplementary Results Sect. 3).

### Compared to older adults, young adults’ mnemonic discrimination showed higher sensitivity to semantic similarity

To address the challenge of testing the effect of fine-grained continuous semantic similarity, which was not possible using d’ values and predefined lure bins, in a final GLMEM we modeled recognition responses with fixed effect of age group (old adults, young adults) in interaction with the continuous cosine similarity metric. The model was defined without any covariate factors, similarly to the simple model. For this analysis we restricted the phrase pool for only target and lure conditions (close and distant) because cosine similarity as a continuous measure is not interpretable in case of foils, since we have no baseline encoded phrase to measure distance. As such, targets are assigned a cosine similarity of 1.0, since they are the exact repeat of a baseline encoded object. This model showed a good fit (*AIC* = 7598.1*, conditional R*^*2*^ = 0.452), and revealed a significant main effect of cosine similarity metric (*estimate* = 158.0*, SE* = 23.3,* p* < 0.001, *CI* = 119.0–211.0), with higher cosine similarity associated with a higher likelihood of ‘old’ responses; a main effect of age group (*estimate* = 3.67, *SE* = 0.77,* p* < 0.001, *CI* = 2.55–5.26), with older adults responding ‘old’ more often than young adults; and a cosine similarity x age group interaction (*estimate* = 0.25, *SE* = 0.5,* p* < 0.001, *CI* = 0.17–0.37) with young adults showing a larger increase in ‘old’ responses as a function of semantic similarity (Fig. 3C).

### Mnemonic discrimination of perceptual lures was positively associated with mnemonic discrimination of conceptual lures in young adults

**Figure 4:**
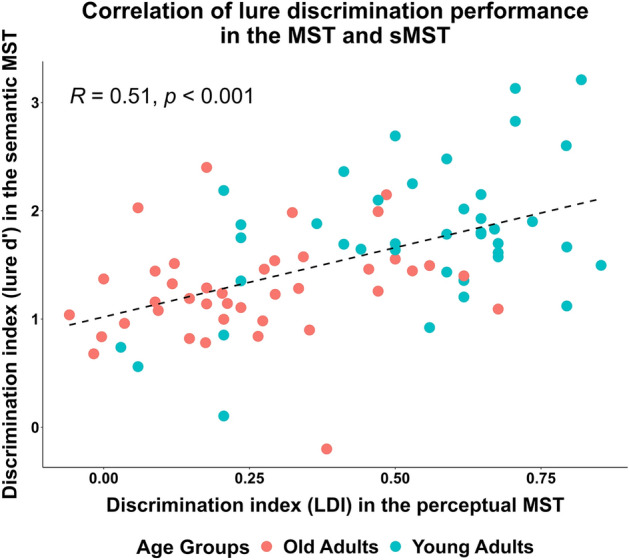
Correlation of lure discrimination performance in the MST and sMST. Correlation of the sMST d’ scores of targets vs. lures collapsed across close and distant bins with the LDI in the original MST, both denoting the discriminability of lures and targets in the recognition phases of the respective tasks. Points show the d’ scores of old adults (red) and young adults (green), while dotted lines show the linear correlation slopes. Correlation was significant in the whole sample (*r* = .51, *p* < .001).

Finally, to explore the relationship between perceptual mnemonic discrimination and conceptual mnemonic discrimination, we used Pearson’s correlation comparing the mnemonic discrimination performance on the sMST and the MST separately for young and older adults. Because in the MST there was no within-subject manipulation of lure similarity, we binned all lures in the sMST into a single category and calculated a lure vs. foil d’ score, that was comparable to the lure discrimination index (LDI) of the MST calculated as the proportion of ‘similar’ responses to foils minus the proportion of ‘similar’ responses to lures^16^. LDI and d’ scores showed a significant positive correlation in the young adult (*r* = 0.46,* p* < 0.001) but not in the older adult (*r* = 0.2,* p* = 0.21) sample. Thus, we looked at whether correlations in our two independent subsamples are significantly different by using a Fisher’s z-test, revealing no significant difference between the two correlations (*z* = 1.28, *95% CI* =  − 0.1386–0.6457, *p* = 0.2). Based on this result, we consider the significant positive correlation in our whole sample (*r* = 0.51, *p* < 0.001) to signify a positive relationship between perceptual and semantic lure discrimination (Fig. 4.).

## Discussion

In this study, we investigated whether age-related differences in the cognitive process of mnemonic discrimination generalize to the discrimination of semantically similar verbal episodic memory traces. To achieve this, we developed the sMST task, which allowed us to test discrimination of incidentally encoded adjective-noun phrases as a function of semantic similarity. We assessed and parametrically manipulated semantic similarity using the Hungarian word2vec word-embedding^37^. To ensure that any detected age-related differences in sMST performance are driven specifically by differences in mnemonic discrimination, we controlled for individual cognitive abilities as well as stimulus properties known to be differentially affected in aging.

Our study has two key findings. First, we showed that mnemonic discrimination of phrases was sensitive to semantic similarity: participants were more likely to false alarm for lures more similar to previously encoded phrases. Second, younger adults outperformed older adults and their discrimination performance showed differential sensitivity to semantic similarity, such that for young adults a marked increase in false alarm rate was observed only at the highest levels of similarity, whereas old adults showed a steady increase in false alarms as a function of similarity.

These latter findings are in line with prior reports of diminished ‘separation bias’, i.e., a bias to identify lures as old, in older compared to younger adults, as well as a differential sensitivity of mnemonic discrimination to perceptual similarity^71,72^. Importantly, our results extend these findings to the domain of conceptual representations, and support the notion that mnemonic discrimination is sensitive to semantic distance and can be regarded as a domain-general cognitive process^73–75^ targeting both perceptual and conceptual representations. The observed correlation between MST and sMST performance in our sample hint at the possibility that mnemonic discrimination of semantically similar episodic memory traces may also rely on the neural operation of hippocampal pattern separation^76–78^. This is consistent with recent propositions on the role of the hippocampus in accessing and manipulating semantic information^5,23,79,80^. Yet, future neuroimaging studies are needed to corroborate this speculation.

The age-related difference in conceptual mnemonic discrimination found in our study contrasts earlier findings by Ly and colleagues^20^ who found no evidence for an age-difference in ‘conceptual pattern separation’. We argue that due to the parametric manipulation introduced, the sMST was sensitive enough to detect this age-effect.

Interestingly, we found that vocabulary—a proxy for crystallized knowledge—had no effect on mnemonic discrimination of semantically similar memory traces. However, fluid abilities such as processing speed for younger adults, and phonological awareness for older adults interacted with age in our complex model (which included covariates), suppressing the age effect. This suggests that age-related-differences may manifest via declining fluid abilities affecting the processing of otherwise relatively intact crystallized representations. This interpretation also fits well with accounts of an age-related shift from specific towards gist-based memories^12,81^, and suggests that specificity of conceptual—and not only perceptual—memory traces is impacted. For instance, compared to young adults, older adults have been shown to be more likely to falsely recall thematically related words (e.g., ‘cold’), when studying a word list (e.g., ‘snow’, ‘warm’, ‘ice’, ‘winter’), suggesting an age-related bias towards the gist rather than the verbatim memories. The ‘fuzzy trace theory’ interprets this effect as evidence for the rapid decay of specific memory traces, and the survival of the less precise ‘fuzzy trace’^18^. Our finding that older adults show more false alarms for lower similarity lures than young adults may be the effect of an increasing decay of verbatim memories, leading to a ‘fuzzy trace’ bias.

The detected age-differences in episodic specificity of conceptual memory representations have implications for understanding age-related changes in accessing representations within an intact semantic memory system. Even though crystallized intelligence and the general knowledge-base remains relatively intact during healthy aging^1–4^, decline in fluid abilities may affect how these semantic memory traces are accessed, encoded and retrieved. However, these effects may only be picked up by sensitive measures of specific cognitive processes, such as the sMST used in our study. This interpretation echoes similar recent behavioral findings^82^ which detected an age-related decline in controlled retrieval of semantic information. Taken together, these findings also further the understanding of age-related changes in episodic access to semantic representations. Several prior studies have detected representational changes in aging that may affect memory specificity^83–85^. Our results add to this by suggesting that changes in processing also affect access of memories across different types of representations. Future studies assessing both representations and operations at the neural level are needed to understand their combined contributions to age-related changes in memory specificity.

Methodologically, our study fits into a broader set of recent studies highlighting the benefit of using word2vec word-embeddings to study neurocognitive mechanisms of memory as a function of semantic similarity^23,86^. For instance using these techniques, a recent study^22^ showed that compared to healthy controls, patients with hippocampal damage were less able to list features distantly related to common concepts (such as ‘leather’ for ‘book’ versus ‘read’ for ‘book’). Others^87^ suggested that this could potentially be interpreted as a lack of hippocampal pattern completion that could guide retrieval of concept-feature associations across semantic spaces.

Finally, a somewhat unexpected result of our study—potentially deserving more attention in future studies—was that older adults false alarmed less for distant lures compared to foils. One possibility is that these semantically distant lures facilitated correct rejection of the lure phrase via the combined presence of two signals: A high familiarity signal of the shared noun, and low familiarity signal of the adjective. In contrast correct rejection of foils may have relied on a single familiarity signal. Another possibility is a conceptual limitation inherent in our definition of distant lures: Distant lures were not always simply more distant from the target than close lures, but sometimes lacked a meaningful connection. Take for example the target phrase ‘exotic zoo’ which has a similar meaning as ‘strange zoo’—the close lure –, whereas ‘public zoo’—the distant lure—has a different meaning altogether. This may have led to distant lures being encoded in gist representations different from the target concept, in turn facilitating target-lure discrimination.

Another limitation of our study was that the corpus of the Hungarian word2vec was limited to the ‘press’ sub-corpus of the HNC^38^. Even after our best efforts to filter these phrases, most of the stimuli has a subjective feeling of coming from a magazine, that we accommodated for by instructing our participants to act as an imagined editor for such a magazine. These biases can be attenuated in future studies by modeling semantic relationship between concepts and features by asking participants to list features for selected concepts and later vectorize them^88^.

## Conclusion

Overall, our study supports the notion of an age-related decline of mnemonic discrimination and extends previous research by showing that this decline is not limited to the perceptual domain and is sensitive to semantic similarity. Our results highlight the importance of investigating specific mnemonic processes within different representational domains matched across task demands^6^. Our results also support the notion that age-related changes in memory may lead to a bias towards generalization and gist-based processing of semantic information in healthy aging, occluding the specific semantic details of episodic memory traces.

### Supplementary Information

Supplementary Information.

## Data Availability

All materials, data, analysis pipelines and processed results are accessible on the Open Science Framework (OSF) repository of this study: https://osf.io/uhmvw/. The study was preregistered at OSF: https://osf.io/62d5p. Preliminary results were presented as a poster at the 22nd conference of the European Society for Cognitive Psychology (ESCOP 2022)—available here: https://osf.io/k65nc. We have no conflict of interest to disclose.
