# Supplementary material for: Discrimination of semantically similar verbal memory traces is affected in healthy aging
Source: Sci Rep. 2024 Aug 2;14:17971. doi: 10.1038/s41598-024-68380-0 (PMC11297280; doi:10.1038/s41598-024-68380-0)
Supplement: Supplementary file 1 — Supplementary Information. [file 41598_2024_68380_MOESM1_ESM.pdf]

Discrimination of semantically similar verbal memory traces is affected in healthy aging

Alex Ilyés, Borbála Paulik, and Attila Keresztes

Supplementary Material

Covariates

Here we present the detailed description of all the covariates we administered in the order of administration:

Montreal Cognitive Assessment (MoCA)

We used the standardized Hungarian MoCA (Nasreddine et al., 2005; Volosin et al., 2013) to screen for dementia in elderly participants only, with a 23-point cut-off.

Non-word repetition task

Participants (Archibald & Gathercole, 2006; Hungarian standardization: Racsmány et al., 2006) were instructed to repeat pre-recorded non-words ranging from one to nine syllables in length presented auditorily through speakers. Participants were presented with blocks of four non-words with syllable length increasing from one to nine across completed blocks. A block was considered completed if the participant correctly repeated two of four non-words. The syllable length of non-words in the last completed block was used as a measure of non-word span. Substitutions within voiced and unvoiced consonant pairs (e.g., substituting 'b' with 'p') were not considered errors. Raw number of repeated words was also registered, offering a more fine-grained scoring that can differentiate participants with the same level of non-word span.

BECK – short version

Participants filled out the short version of the Beck Depression Inventory (Beck et al., 1974; Hungarian standardization: Rózsa et al., 2001) containing nine statements about their feeling

and mood in the month preceding data collection. They were asked to indicate how well the statements described their feelings on a 1–4 Likert scale where 1 meant ‘not at all’ and 4 meant ‘all the time’. The sum of the points given was used as a depression score.

Health and Lifestyle Questionnaire

Participants filled out an in-house questionnaire on health and lifestyle (see Supplementary Table 1) assessing variables frequently related to age-related memory decline (Aghjayan et al., 2022; Bruce et al., 2008; Kempermann, 2019; Larson et al., 2006; Maguire et al., 2000; Mårtensson et al., 2012; Rovio et al., 2005; Woollett & Maguire, 2011). These included amount and frequency of alcohol, nicotine and caffeine consumption both 24 hours before data collection and regularly, number of hours of sleep on the preceding and an average night, and the hours spent sitting on an average day, as well as the amount, frequency and duration of active, regular sports done by the participant in the last 10 years.

Mnemonic Similarity Task

In this task (Kirwan & Stark, 2007; Stark et al., 2013) participants saw 64 pictures of everyday objects in an incidental encoding phase and were instructed to decide whether the depicted objects are used ‘indoor’ or ‘outdoor’. In each trial, a fixation cross appeared for one second, followed by an object in the middle of the screen for two seconds. In a recognition phase administered after a two-minute break, participants saw 96 pictures of everyday object with 32 targets (pictures identical to pictures of objects seen at encoding), 32 lures (i.e., similar pictures similar to pictures of objects seen at encoding), and 32 foils (new pictures of novel objects), and were instructed to indicate if the objects were ‘old’, ‘similar’, or ‘new’. In each trial, a fixation cross appeared for one second, followed by an object in the middle of the screen for four seconds. For both encoding and recognition, participants responded via button presses on a QWERTY-keyboard.

It is important to note that the MST procedure we followed is an incidental task, however our participants already completed the sMST, thus probably had an expectation about this surprise memory test. This however should not alter the performance markedly, as shown by Stark et al. (2015)

WAIS – Vocabulary Test

In this subtest of the Wechsler Adult Intelligence Scale (WAIS-IV; Wechsler, 2008, Hungarian adaptation: Rózsa et al., 2010), participants were instructed to describe the meaning of a series of 27 Hungarian words. The experimenter rated responses for each word on a scale of 0–2, and concluded the test if participants scored 0 for three consecutive words. A final score with a maximum of 54 gave a proxy for the vocabulary of a participant.

WAIS – Digit-Symbol Substitution Test

In this subtest of the WAIS, participants received a sheet with 135 digits printed in 15 rows from left to right in a horizontal layout. Below each digit there was a blank box. Each digit from 1-9 corresponded to a unique symbol printed at the top of the paper. Participants were instructed to copy the corresponding symbol into the blank box below each digit, proceeding one digit at a time from left to right, without correcting errors. The final score was the sum of all symbols correctly drawn within two minutes.

Randomization

Here we provide further detail on our randomization process:

To ensure that each stimulus in each condition is equally distributed across lists despite different number of stimuli per condition, we constructed two phrase pools: In the target

phrase pool each phrase had three versions (target, close lure, and distant lure). In the foil phrase pool, each phrase had only one version.

During randomization, we made sure that all phrases from the target phrase pool were presented an equal number of times as target, close lures, and distant lures, and that all phrases from the foil phrase pool were presented as fillers at encoding or foils at recognition the same number of times. As each of the 100 phrases in the target phrase pool had both close and distant lure versions, and in a recognition phase 25 close and 25 distant lures were presented, we presented all 100 close and 100 distant lures once over four consecutive lists to four different participants. Phrases in the recognition phase were separated into four groups of 25 phrases: For the first list (presented to the first participant), group 1 were close lures, group 2 were distant lures and group 3 and 4 were targets, for the second list (presented to the second participant), group 2 are close lures, group 3 were distant lures and group 4 and 1 are target repeats, and so on. This guaranteed that across four lists, for all phrases, we presented a close and a distant lure version once, and target versions twice.

We also made sure that all 125 phrases from the foil phrase pool were presented as foils at recognition equal number of times. In a single list we used all phrases from this phrase pool, but only 25 phrases functioned as foils at recognition, the rest of the 100 phrases all functioned as fillers at encoding. Thus, over five consecutive lists, all phrases from the foil phrase pool were used as foils exactly once. As a result, 40 participants provided 10 data points for each close and distant lure, 20 data points for each target, and 8 data points for each foil.

Supplementary Results

1.Younger adults made more accurate and faster mnemonic decisions.

We analyzed age-differences in reaction times, overall accuracy, and response rates in both encoding and recognition of the sMST. Overall accuracy in the next two sections refers to the rate of correct responses given by participants on the task independent of semantic similarity. Reaction times were significantly different between the two age groups only in the recognition phase (Supplementary Figure 1), with young (M = 1.45 sec, SD = 0.3 sec) adults being faster than older (M = 1.74 sec, SD = 0.45 sec) adults ($t\ (78) = 6.79$, $p < .001$). Overall accuracy during recognition was higher for young (M = .81, SD = .09) adults compared to older (M = .73, SD = .08) adults ($t\ (76.971) = -4.06$, $p < .001$). Finally, in the encoding phase (Supplementary Figure 2), young (M = .74, SD = .12) adults made fewer overall ‘good’ judgements (i.e., accepted fewer phrases as imaginary editors), than older (M = .82, SD = .09) adults ($t\ (71.359) = 3.34$, $p = .001$). No other differences were significant.

2. Recognition memory was independent of incidental encoding responses.

To test whether participants’ subjective judgments during encoding had any effect on recognition performance, we compared the overall accuracy on the task for phrases that were previously judged as ‘good’ (M = .75, SD = .17) vs. ‘not good’ (M = .78, SD = .21) using an independent sample t-test and found no significant difference ($t\ (428.42) = -1.21$, $p = .23$). Further, a two-way mixed ANOVA with age as a between-subject factor and encoding response as a within-subject variable revealed neither a main effect of encoding response ($F\ (1, 458) = 1.54$, $p = .22$), nor an interaction ($F\ (1, 458) = 1.71$, $p = .19$).

3. Further results of the complex GLMEM (Results 5 continued)

We defined a complex model, with stimulus condition and age group in interaction and covariates as interaction terms with their respective fixed effects (participant-level covariates

with age group, noun frequency with stimulus condition, and psycholinguistic item-level covariates in a three-way interaction with both stimulus condition and age group, Figure 3/B).

A look at interactions of item-level covariates revealed a significant targets x noun frequency (estimate = .93, SE = .03, $p = .01$, CI = .87 – .98) and a foils x noun frequency (estimate = 1.08, SE = .04, $p = .02$, CI = 1.01 – 1.15) interaction indicating that participants were more likely to respond erroneously for both target and foil phrases.

Finally, we found significant three-way interactions of close lure x young adults x old adult score of concreteness (estimate = .81, SE = .08, $p = .04$, CI = .66 – 1.0), close lure x young adults x young adult score of concreteness (estimate = 1.47, SE = .15, $p < .001$, CI = 1.21 – 1.79), and distant lure x young adults x young adult score of concreteness (estimate = 1.3, SE = .13, $p < .01$, CI = 1.07 – 1.58), indicating that young adults were more likely to false alarm for both close and distant lures as the concreteness measured by a separate sample of young adults of phrases increased, yet they were less likely to false alarm based on increasing concreteness measures determined by a separate sample of old adults in case of close lures. Interestingly, a significant close lure x old adults x young adult score of concreteness (estimate = 1.2, SE = .11, $p = .04$, CI = 1.01 – 1.44) showed that old adults were more likely to falsely recognize close lures as the concreteness measured by young adults increased, but there were no effect of the ratings by a separate sample of old adults (estimate = .92, SE = .09, $p = .37$, CI = .77 – 1.11).

Supplementary Figures and Tables

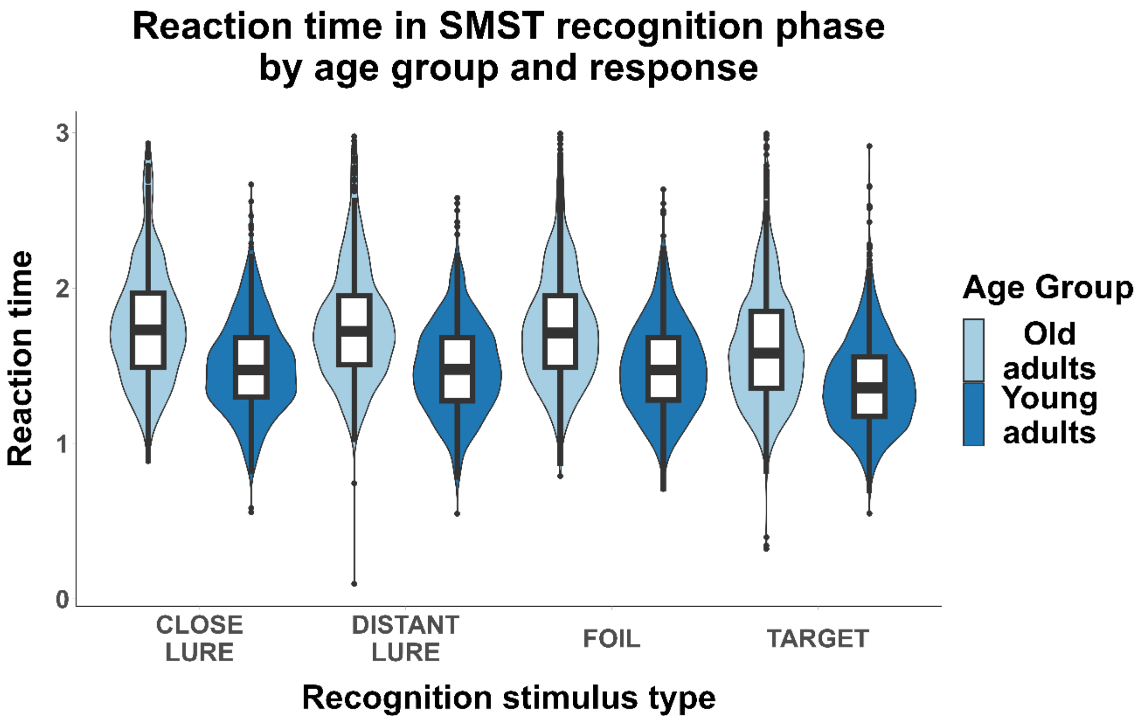

Supplementary Figure 1. Reaction times in sMST recognition phase.

A significant difference in reaction time was found in the recognition phase of the sMST. Young adults were faster than older adults in all conditions. The x-axis shows stimulus types at recognition, the y-axis shows reaction time in seconds. Light shade violin plots show old adult reaction times, while dark shade violin plots show young adult reaction times.

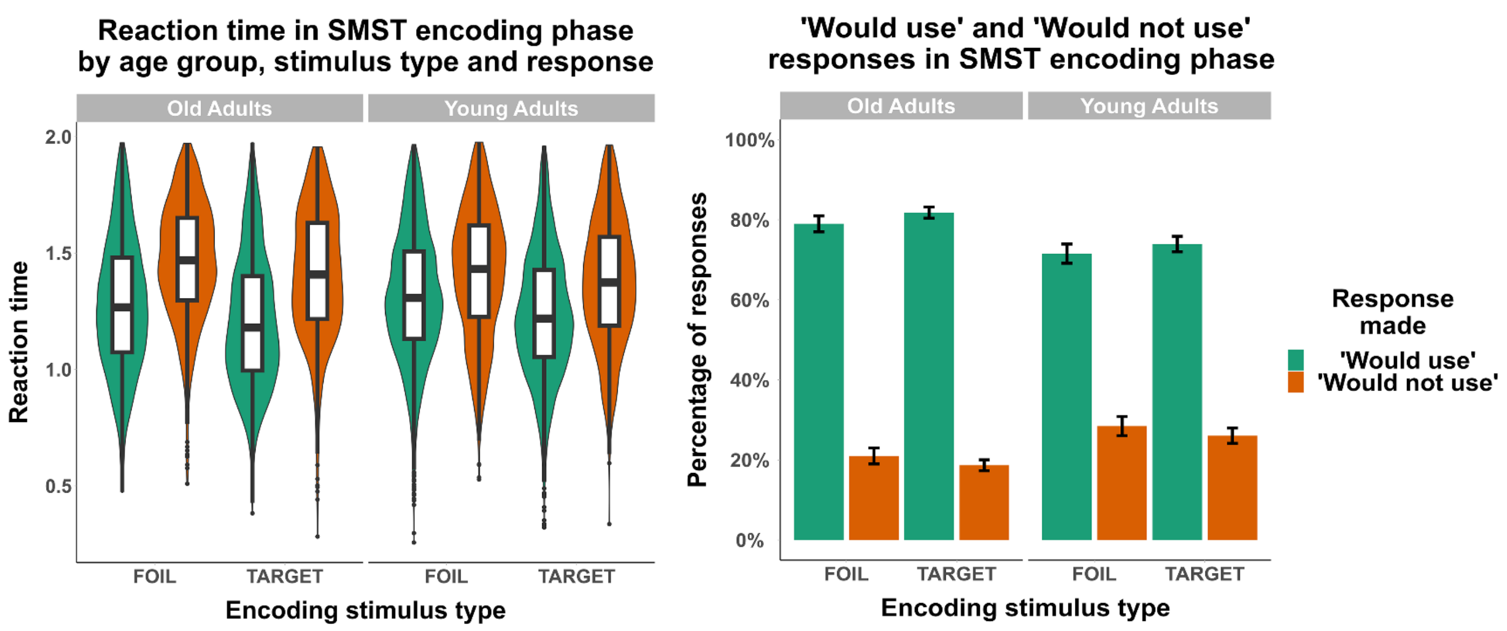

Supplementary Figure 2. Results from sMST encoding phase.

Reaction times (left) and response rates (right) in the encoding phase. The x-axis shows the different stimulus types, while the y-axis shows reaction time in seconds (left) and the percentage of given responses (right). Error bars represent the standard error.

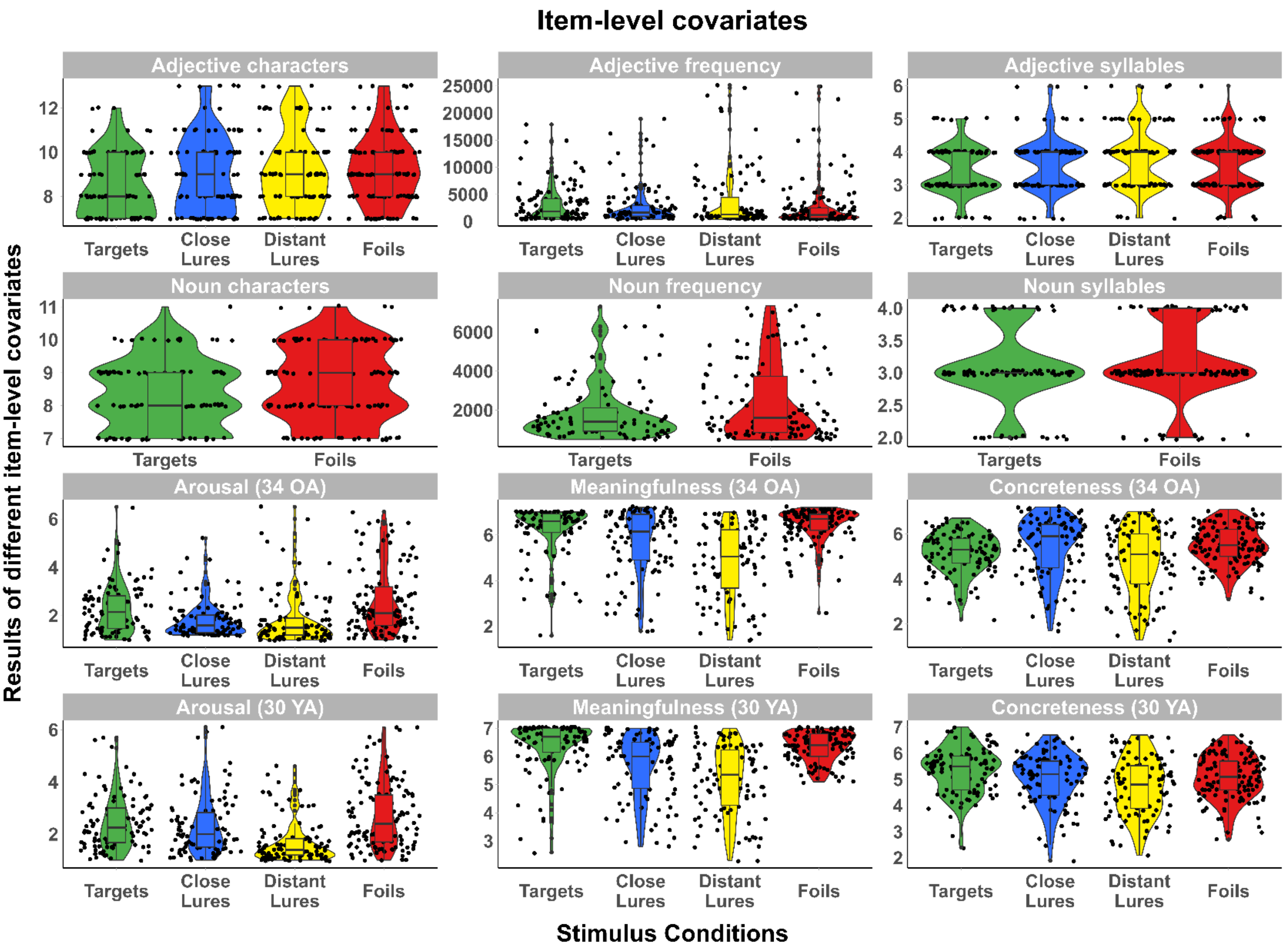

Supplementary Figure 3. Scores of item-level covariates.

Frequency is gathered from the HNC [49], character count and syllable count are measured for both adjectives and nouns. Concreteness, meaningfulness, and arousal data were collected in a questionnaire with 1-7 Likert scales for each measure. Questionnaires were administered on 30 university students (M_{age} = 22.56, *SD* = 6.37, $M_{education}$ = 14.15, females = 24, all Caucasian) and 34 older adults (M_{age} = 71.18, *SD* = 5.1, $M_{education}$ = 18.15, females = 18, all Caucasian) for 1000 HUF vouchers, and this resulted in 10 data points on each scale for each word in both age group.

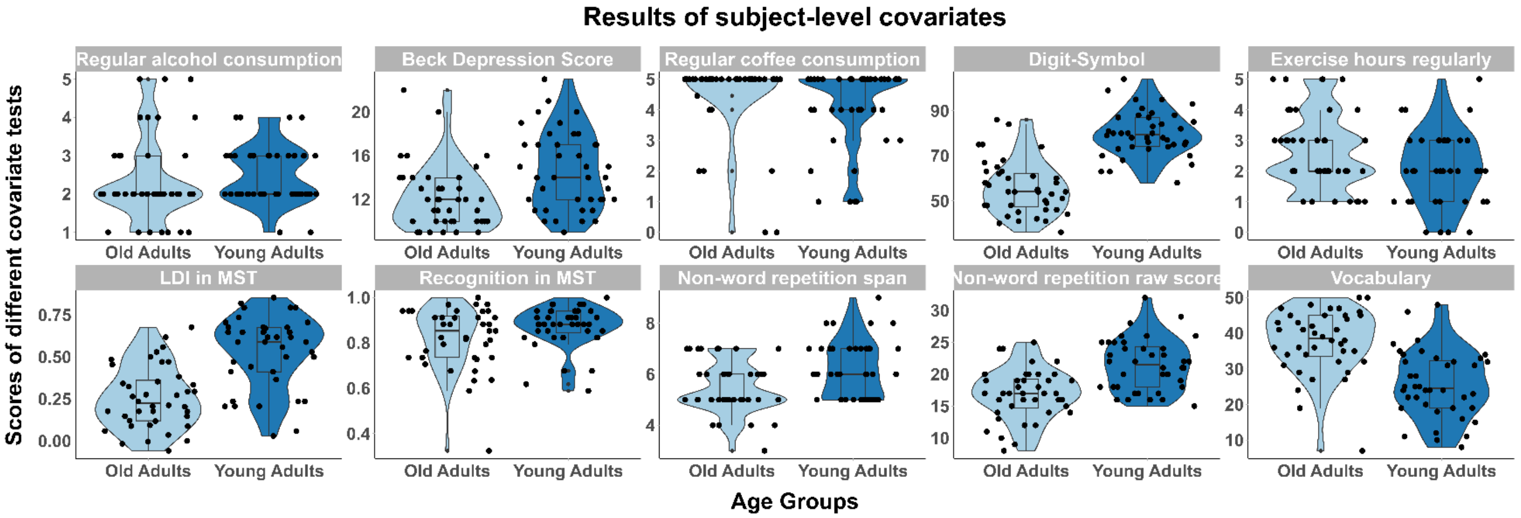

Supplementary Figure 4. Scores of participant-level covariates.

Regular alcohol and coffee consumption was surveyed on a 5-level Likert scale (never, rarely, sometimes, often, always). Depression score is the sum of points given on the Beck Short Inventory for 9 items on a 4-level Likert scale. Digit-Symbol measures processing speed based on symbols drawn by hand under respective numeric fields. LDI and Recognition Scores are derived from the original MST with picture stimuli. Non-word repetition score shows the number of words repeated by the participants, while non-word span is the last count of syllables completed by them. Vocabulary is measured by the WAIS subtest, where participants define increasingly rare expressions.

Questions	Young adults (n=40)	Old adults (n=40)
Have you consumed any alcohol in the last 24 hours?	32.5% yes	30% yes
How many units of alcohol have you consumed?	*M* = 2.33, *SD* = 1.83	*M* = 1.36, *SD* = 0.81
How often do you consume alcohol? (1 = Never, 2 = Only on special occasions, 3 = Once a week, 4 = 3-5 time a week, 5 = Everyday)	*Median* = 2	*Median* = 2
How many units of alcohol do you usually consume?	*M* = 3, *SD* = 1.25	*M* = 1.71, *SD* = 0.81
Have you consumed any caffeine in the last 24 hours?	65% yes	85% yes
How many units of caffeine have you consumed?	*M* = 1.12, *SD* = 0.33	*M* = 1.21, *SD* = 0.64
What types of caffeine do you usually consume?	Coffee	Coffee
How often do you consume caffeine? (1 = Never, 2 = Only on special occasions, 3 = Once a week, 4 = 3-5 time a week, 5 = Everyday)	*Median* = 5	*Median* = 5
How many units of caffeine do you usually consume?	*M* = 1.51, *SD* = 0.56	*M* = 2.03, *SD* = 1.07
Have you consumed any nicotine in the last 24 hours?	5.9% yes	13.8% yes
How many units of nicotine have you consumed?	5 (1 person)	1 (4 person)
What types of nicotine do you usually consume?	cigarette	cigarette
How often do you consume nicotine? (1 = Never, 2 = Only on special occasions, 3 = Once a week, 4 = 3-5 time a week, 5 = Everyday)	*Median* = 1	*Median* = 1
How many units of nicotine do you usually consume?	*M* = 2.3, *SD* = 1.14	*M* = 12.5, *SD* = 6.45
How many hours do you usually sleep? (1 = Less, than 5 hours, 2 = 5-6 hours, 3 = 6-7 hours, 4 = 7-8 hours, 5 = More than 8 hours)	*Median* = 4	*Median* = 3
How many hours have you slept last night? (1 = Less, than 5 hours, 2 = 5-6 hours, 3 = 6-7 hours, 4 = 7-8 hours, 5 = More than 8 hours)	*Median* = 4	*Median* = 3
Are you exercising regularly?	85% yes	90% yes
How often do you exercise? (1 = Less than 2 hours a week, 2 = 2-5 hours a week, 3 = 5-8 hours a week, 4 = 8-10 hours a week, 5 = More than 10 hours a week)	*Median* = 2	*Median* = 2
How many hours do you spend sitting on a regular day?	*M* = 7.29, *SD* = 2.88	*M* = 5.97, *SD* = 2.89

Supplementary Table 1. Questions of our in-house health and lifestyle questionnaire.

Questions were administered in the order given here. Where a predetermined scale was used, response options are indicated in brackets. We provide descriptive statistics of both young adults and old adults on the questions. The actual questionnaire is available on our OSF page: https://osf.io/uhmvw/.

	Arousal scale				Concreteness scale				Meaningfulness scale			
	df	***F***	***p***	**η^2**	***df***	***F***	***p***	**η^2**	***df***	***F***	***p***	**η^2**
**Age group**	*1, 842*	*3.113*	.078	.004	*1, 842*	*7.049*	.008	.008	*1, 842*	*0.873*	.351	.001
**Condition**	*3, 842*	*25.456*	<.001	.083	*3, 842*	*12.340*	<.001	.042	*3, 842*	*72.743*	<.001	.206
**Age group x condition**	*3, 842*	*3.082*	.027	.011	*3, 842*	*4.032*	.007	.014	*3, 842*	*1.766*	.152	.006

Supplementary Table 2. Results of two-way ANOVAs of each item-level covariate scores by the interaction of age group and phrase condition.

Arousal, meaningfulness, and concreteness of phrases were assessed by an independent sample of 30 younger adults and 34 older adults. Three two-way ANOVA was done with the covariate scores as dependent variables, and phrase condition in interaction with age group as independent variables. Note, that these tests do not necessarily reveal confounding effects on our memory tests, as shown by the GLMEM (more details in section 5. Of the Results), where we included each measure as a covariate.
